# Volcanic dust veils from sixth century tree-ring isotopes linked to reduced irradiance, primary production and human health

**DOI:** 10.1038/s41598-018-19760-w

**Published:** 2018-01-22

**Authors:** Samuli Helama, Laura Arppe, Joonas Uusitalo, Jari Holopainen, Hanna M. Mäkelä, Harri Mäkinen, Kari Mielikäinen, Pekka Nöjd, Raimo Sutinen, Jussi-Pekka Taavitsainen, Mauri Timonen, Markku Oinonen

**Affiliations:** 1 Natural Resources Institute Finland, Eteläranta 55, Rovaniemi, Finland; 2 Laboratory of Chronology, Finnish Museum of Natural History, Gustaf Hällströmin katu 2, University of Helsinki, Helsinki, Finland; 3 Department of Geographical and Historical Studies, Yliopistokatu 7, University of Eastern Finland, Joensuu, Finland; 4 Finnish Meteorological Institute, Erik Palménin aukio 1, Helsinki, Finland; 5 Natural Resources Institute Finland, Tietotie 2, Espoo, Finland; 6 Geological Survey of Finland, Lähteentie 2, Rovaniemi, Finland; 7 Department of Archaeology, Henrikinkatu 2, University of Turku, Turku, Finland

## Abstract

The large volcanic eruptions of AD 536 and 540 led to climate cooling and contributed to hardships of Late Antiquity societies throughout Eurasia, and triggered a major environmental event in the historical Roman Empire. Our set of stable carbon isotope records from subfossil tree rings demonstrates a strong negative excursion in AD 536 and 541–544. Modern data from these sites show that carbon isotope variations are driven by solar radiation. A model based on sixth century isotopes reconstruct an irradiance anomaly for AD 536 and 541–544 of nearly three standard deviations below the mean value based on modern data. This anomaly can be explained by a volcanic dust veil reducing solar radiation and thus primary production threatening food security over a multitude of years. We offer a hypothesis that persistently low irradiance contributed to remarkably simultaneous outbreaks of famine and Justinianic plague in the eastern Roman Empire with adverse effects on crop production and photosynthesis of the vitamin D in human skin and thus, collectively, human health. Our results provide a hitherto unstudied proxy for exploring the mechanisms of ‘volcanic summers’ to demonstrate the post-eruption deficiencies in sunlight and to explain the human consequences during such calamity years.

## Introduction

Explosive volcanic eruptions constitute a natural, external climatic forcing factor when their sulfate aerosol emissions reach the stratosphere and reflect solar irradiance. Short-term impacts of this radiative forcing are well documented over the modern instrumental period and lead to post-eruption cooling of global summer temperatures and reduce the amount of sunlight reaching the biosphere^1^. Tree-ring proxies constitute primary evidence for detecting the magnitudes of volcanic impacts prior to instrumental observations^2^. These annual data provide a precise record of climate anomalies and offer a way to examine the interaction of preindustrial society, climate change and other natural phenomena such as volcanic eruptions^3,4^. In addition to palaeoclimate data derived from tree-ring width/density chronologies, the long history of volcanism can be traced from deep ice cores^5^. Much interest on past volcanic impacts has centered on the mid-sixth century AD climate anomalies^6–12^, with recent proliferation of new data from the bipolar ice-core timescales and sulfur records.

The revised ice-core data reconstruct the radiative forcing from eruptions in AD 536 and 540 and imply that a combined volcanic signal from multiple North American eruptions preceded the AD 536 cooling event, whereas a second cooling phase from AD 541 until 550 was likely due to tropical eruptions^2^. Climate modelling indicates that the decadal radiative forcing of these events totaled larger than that of any other volcanic event in extra-tropical Northern Hemisphere in the last 1200 years, with longer duration of sulfate deposition from the second event^13^. Similar evidence available from high-resolution palaeoclimate data demonstrate climatic cooling on decadal and similar spatial scales^12,14^. Climate anomalies during these years have been linked to crop failure, high number of famines and societal crises in Eurasia^15,16^ and debated as the cause of several large human migrations in the Palearctic^4^.

Topically, the mid-sixth century cooling is suggested to have decreased the agricultural production in Europe, especially at high altitudes and latitudes^13^ where historical crop yields were temperature driven^17,18^. However, this effect appears more subtle over central and southern Europe^13^ where the primary production is in fact more constrained by solar radiation^19^ rather than temperature. As a consequence, the volcanic cooling may not constitute the only climatic factor to explain the human consequences and their nutritional background. Given that terrestrial photosynthesis is strongly limited by irradiance^20^, the human utilization of plant products ought to be similarly affected by post-eruption radiative forcing. It is essential that the AD 536 eruption is believed to have created a ‘dust veil’ that, according to historical accounts from the Mediterranean to eastern Asia, dimmed the sun for more than a year^6–11^. In addition to cooling global temperatures, the dust veil must have dramatically reduced irradiance and thereby photosynthetic products and their contemporary human utilization as a component of the post-eruption forcing. Clearly, quantifying this relationship would help to assess the impact of the explosive volcanic eruptions of AD 536 and 540 on human populations of Europe and elsewhere^3,4,15,16^.

**Figure 1:**
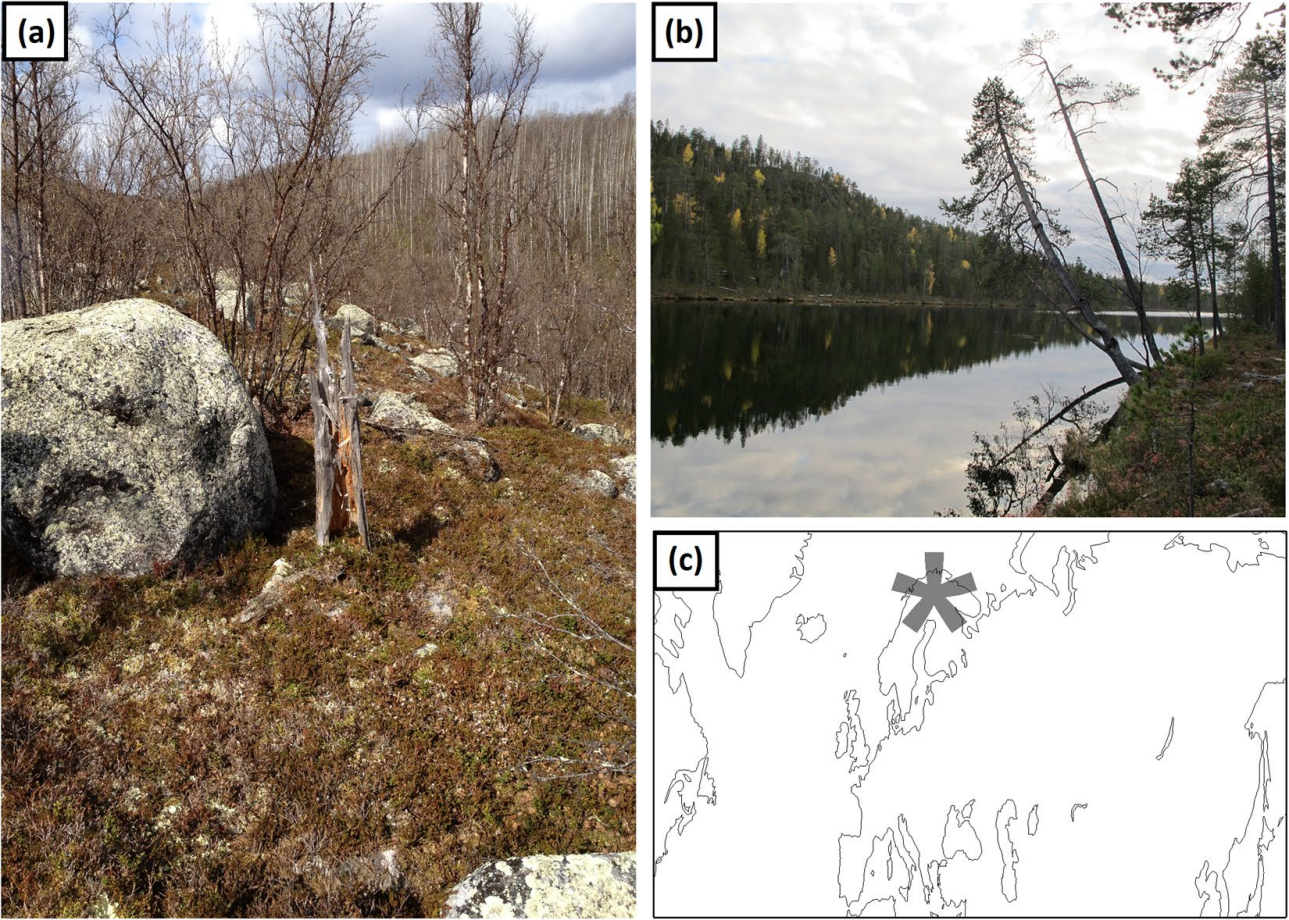
Sampling sites. Carbon isotope data of this study was collected from (**a**) upland and (**b**) riparian settings where the subfossil specimens are preserved in the lacustrine sediment and subaerially, respectively. Map showing the sampling sites for this study (star) (**c**). Photos by Hannu Herva (**a**) and Samuli Helama (**b**). The map was created by OriginPro 2017 SR1 software (http://www.originlab.com/).

In addition to temperature inferences traditionally obtained from tree-ring width/density data, irradiance proxies are obtained from the stable carbon isotope ^13^C/^12^C ratio in tree rings. This ratio (δ^13^C) is explained by the concentration of CO_2_ in the intercellular spaces in the leaf/needle, in relation to its concentration in the air, the ratio between the two varying through photosynthetic gas exchange as controlled by stomatal openings/closure and/or rate of CO_2_ assimilation by the plant^21^. Delineated by the Farquhar model of photosynthesis^22,23^, the δ^13^C is described to increase by high light intensity and/or moisture deficit, whereas the reductions in available light and water surplus lead to decreased δ^13^C. This being the case, the tree-ring samples taken from sites unlikely to suffer from drought ought to contain strong signals of irradiance, such conditions commonly prevailing near the northern timberline^24–27^. Further, the signal strength can be warranted by site selection for sampling the trees from riparian habitats where plants should not suffer from water deficits. To test the hypothesis of reduced irradiance during and after AD 536, we present tree-ring δ^13^C proxy data from subfossil and living pines (*Pinus sylvestris* L.) collected from timberline sites in northern Europe (Fig. 1). These samples represent a composite of sites (Table S1) from the region where the δ^13^C signal remains consistently sensitive to sunlight intensity during the summer (June–August) growing season^27^.

Calibrated against the instrumental records of irradiance, the modern δ^13^C data provide a model to reconstruct the signal over the mid-sixth century ad. Analysing the subfossil δ^13^C assemblage from riparian/upland sites we disentangle the irradiance-dependent δ^13^C variability from any remnant of drought signal. Our high-resolution assessment of the volcanic dust veils shows distinct, well-dated anomaly consistent with evidence in historical–documentary sources, to highlight irradiance as a new palaeoclimate parameter. We show that carbon isotopes of ancient wood offer a reliable proxy of the forcing event, as dated reliably on a calendric timeline analogous to historical records.

## Results

### Subfossil δ^13^C assemblage

**Figure 2:**
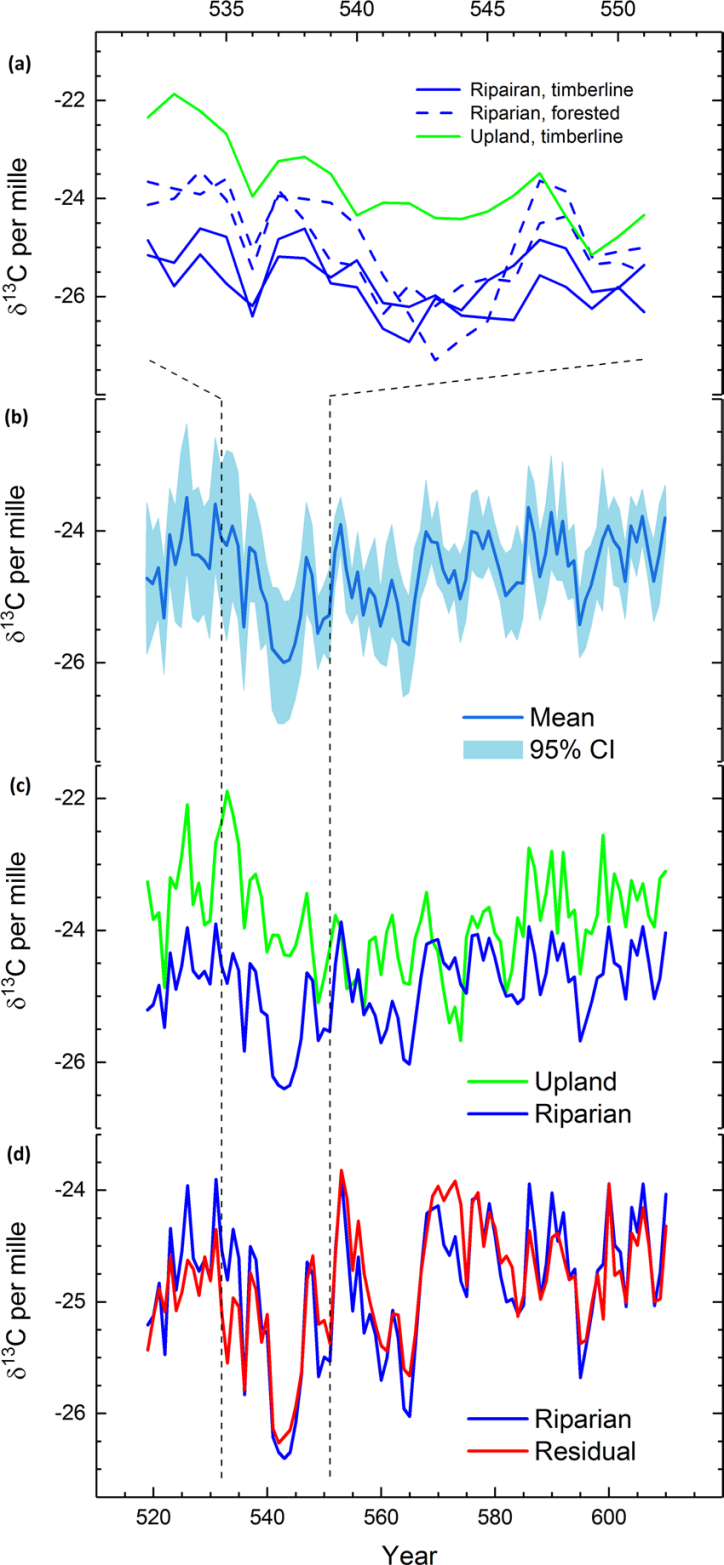
Stable carbon isotope anomalies during the volcanic events. Volcanic signals in AD 536 and 541–544 in carbon isotope ratios (δ^13^C) from the subfossil tree rings with their respective origin (**a**). Mean δ^13^C chronology of all five sample series with 95% confidence interval (**b**), comparison of the riparian δ^13^C chronology and the upland δ^13^C series (**c**), the new, residual δ^13^C series compared with the riparian δ^13^C chronology (**d**). Chronologies shown for interval with δ^13^C data from at least four subfossil pines (AD 519–610).

Averaged chronology of subfossil δ^13^C series pinpoint the AD 536 event as a sharply negative excursion of 0.5–1.6 per mille (‰), followed by a 1–2 year recovery to pre-event δ^13^C values (Fig. 2). The second anomaly starts in AD 541 and is described by a more markedly negative departure with a sustained δ^13^C downturn of 1.6‰ until AD 544 ad. In AD 545 the mean δ^13^C exceeds the value of AD 541. Overall, the δ^13^C series show a strong correlation in their inter-annual variability during the suggested volcanic forcing (Fig. S1). A closer look at the δ^13^C series illustrates markedly negative δ^13^C for riparian samples (Fig. 2A) and most strongly for a tree from forested site during the event. These data exhibit the most distinctive volcanic signals according to their anomalous values. The strong signal in the riparian δ^13^C data contrasts with the upland δ^13^C series having the least depleted δ^13^C values, suggesting a relatively stronger dependence on soil moisture in upland data.

Based on these results and the photosynthesis model^22,23^, we first establish a palaeoecological interpretation for the δ^13^C event. That trees from forested site tend to exhibit most anomalous δ^13^C excursion likely indicates that such shaded individuals were those most stressed during the event. A large magnitude of the signal implies that shading from adjacent trees amplified the effects of reduced irradiance, leading to strongly negative δ^13^C values, in comparison to forest line sites where trees grow more widely spaced. The upland data appears least affected implying that the δ^13^C response was not reinforced by moisture anomalies as the combination of low irradiance and excess of available water had likely intensified the depletion of δ^13^C values in that habitat. Collectively, these findings point towards a dry fog event, such as a volcanic dust veil, as origin of the δ^13^C anomaly in that the conditions responsible for the event represent a combination of reduced irradiance without any noticeable change in hydroclimate.

Further validating the signals, the data of riparian chronology is regressed against the data of upland series, and the residuals from that model are retained as a new δ^13^C series (Fig. 2C), thus being statistically independent of the moisture effects (assumed to be present in upland data). We note that also the new, residual δ^13^C series illustrates an isotopic excursion in AD 536 and between AD 541 and 544 (Fig. 2D). The persistence of such anomalies adds credibility to the dry fog/dust veil assumption and thus to identify the anomaly as a volcanic signal.

### Irradiance reconstruction

**Figure 3:**
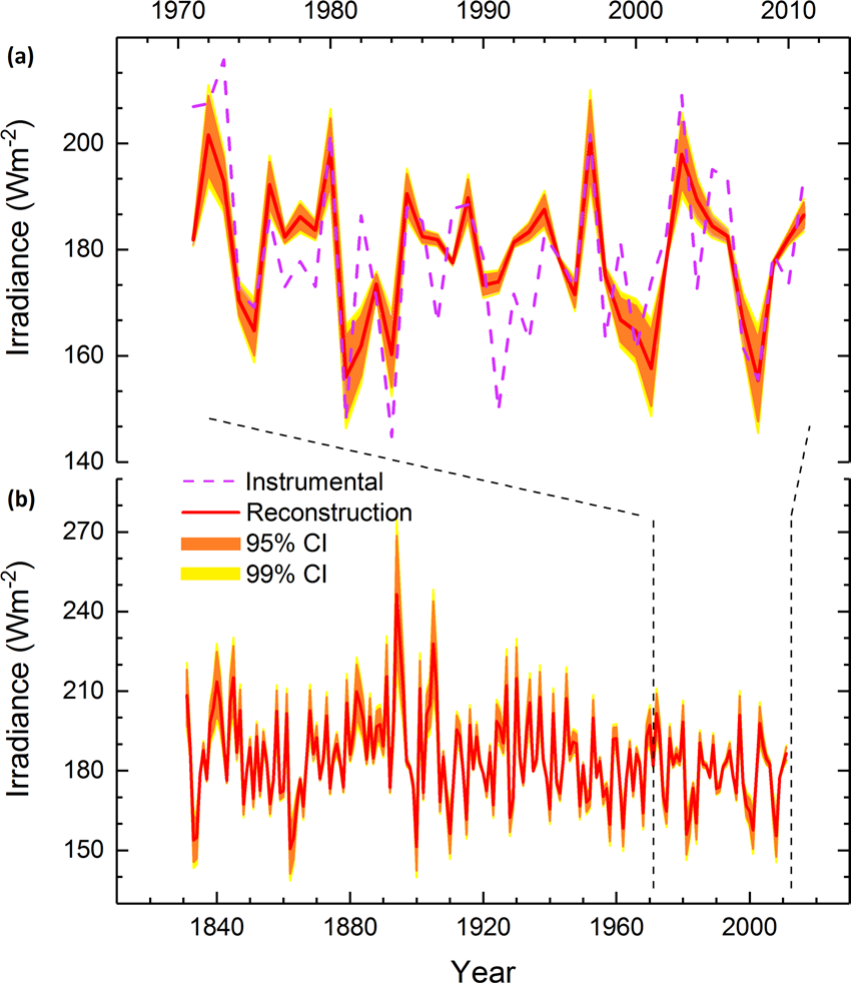
Reconstruction of irradiance. The δ^13^C based estimates (global radiation) (red line) with Monte Carlo^68^ based estimates of 95% (orange area) and 99% confidence (yellow area) intervals (CI) compared with the instrumentally-measured record of global radiation (dashed lilac line) illustrated over the common period (AD 1971–2011) (**a**) and since 1831 when the δ^13^C chronology is covered using at least four pines (**b**). Calibration and verification statistics are detailed in Table S2.

The post- AD 536 irradiance anomalies can be reconstructed using a model calibrated with the modern δ^13^C record. These data provide an estimate of isotopic variance associated with irradiance measured over recent decades by twentieth-century technology (Fig. 3). The final reconstruction model (i.e., the transfer function built over the 1971–2011 period) explain more than half of the variance in the instrumental data (R^2^ = 0.522, p < 0.0001). Thus, our δ^13^C dataset exceeds the thresholds previously set for an acceptable δ^13^C proxy^28,29^. Furthermore, jack-knifed simulations of the original data retain statistically significant (p < 0.01) results and confirm the proxy-to-target agreement for a variable dataset over the instrumental period (Table S2).

**Figure 4:**
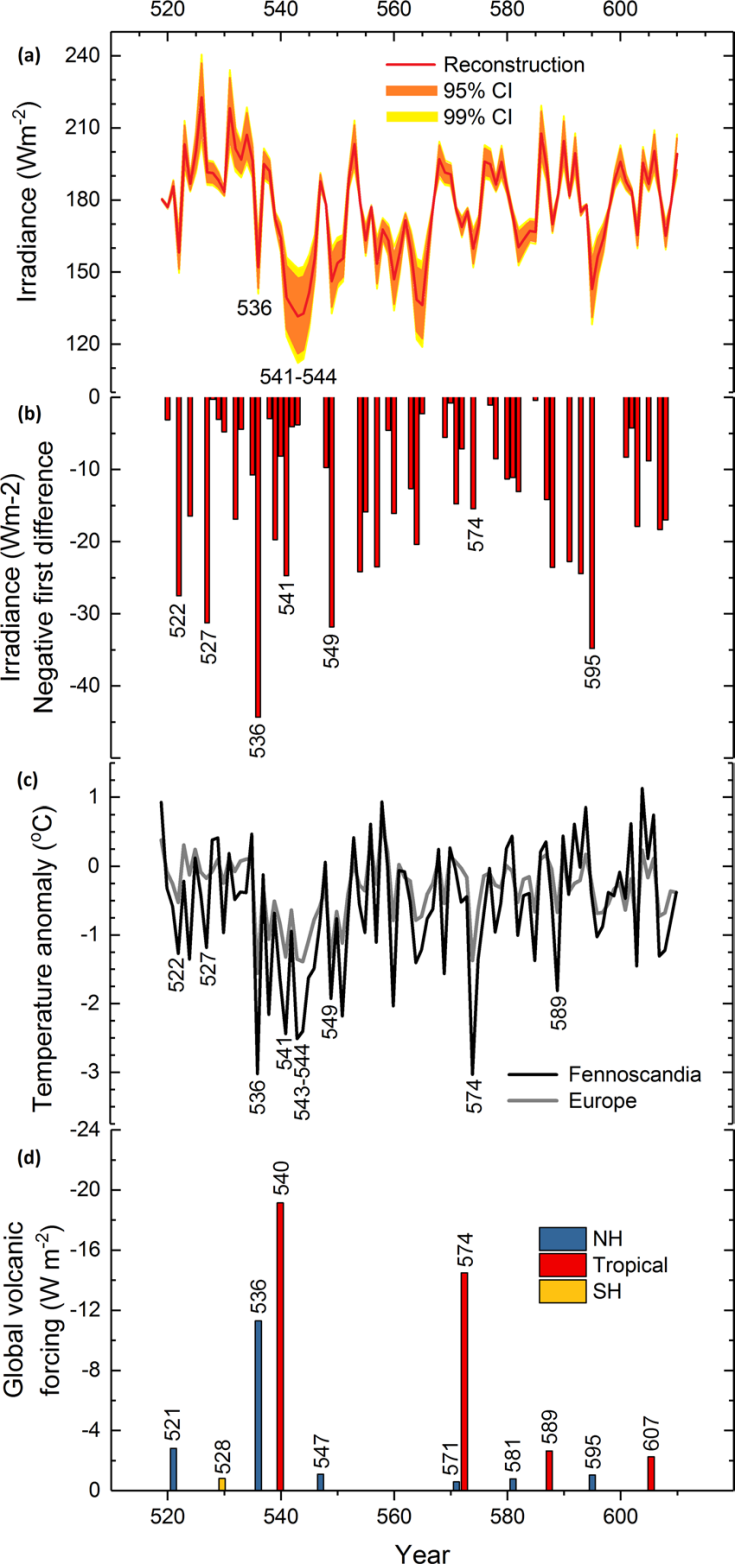
Palaeoclimate reconstructions. Tree-ring δ^13^C based reconstruction of irradiance (global radiation) (red line) with Monte Carlo^68^ based estimates of 95% (orange area) and 99% confidence (yellow area) intervals showing the reduction in irradiance in AD 536 and 541–546 (**a**). Negative first difference of the reconstructed irradiance recording the change in irradiance from previous to concurrent year (**c**). European^36^ and northern Fennoscandian summer (June–August) temperature reconstructions^37^ relative to the AD 1961–1990 baseline (**c**). Volcanic aerosol forcing demonstrating strong AD 536 and 540 events from ice core evidence with list of eruptions of Northern Hemisphere (NH), tropical and Southern Hemisphere (SH) origin over sixth century^2^ (**d**).

**Figure 5:**
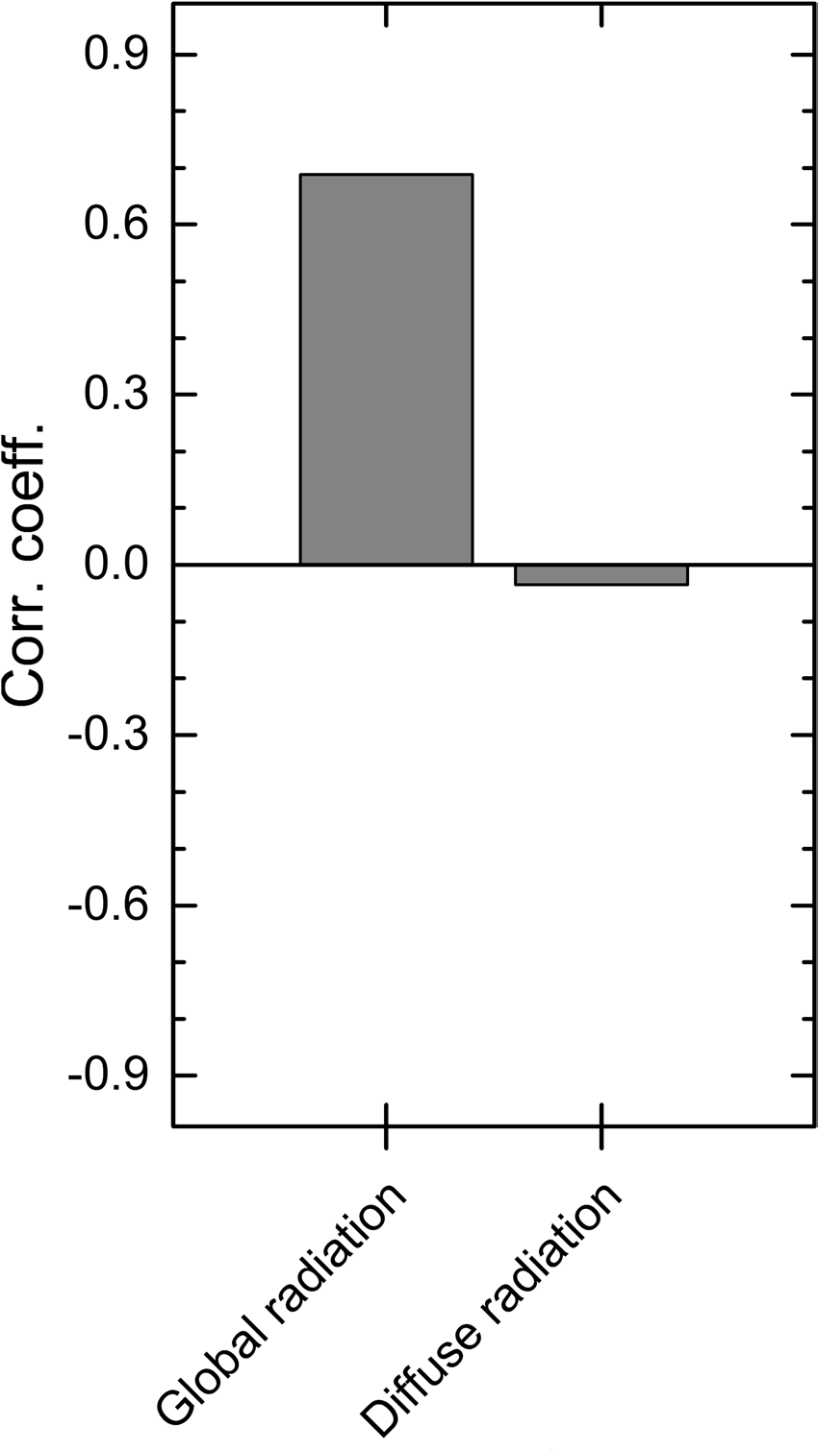
Global radiation and diffuse radiation. Correlations between the modern δ^13^C chronology and records of global radiation and diffuse radiation (AD 1991–2011) observed at the Sodankylä meteorological station in northern Finland.

The δ^13^C-based reconstruction quantifies the strongly reduced irradiance in 536 and especially AD 541–544 (Fig. 4). These findings contrast with the suggestion of enhanced photosynthesis, as suggested following the worldwide increase of diffuse radiation due to volcanic aerosols released during the AD 1991 Pinatubo eruption^30^. We also find no correlation between the δ^13^C and diffuse radiation data measured with instruments during recent decades (Fig. 5). Collectively, these data agree with the tree-ring observations from multiple sites in the Northern Hemisphere (AD 1300–1950) (ref.^31^) and suggest that the effects of overall light loss for photosynthesis have not been compensated by aerosol-driven changes in light composition during volcanic events. Thus, our results concur with biogeochemical models that have explained the enhanced post-Pinatubo CO_2_ sink by several other (non-photosynthetic) land and ocean sink mechanisms^32,33^.

**Figure 6:**
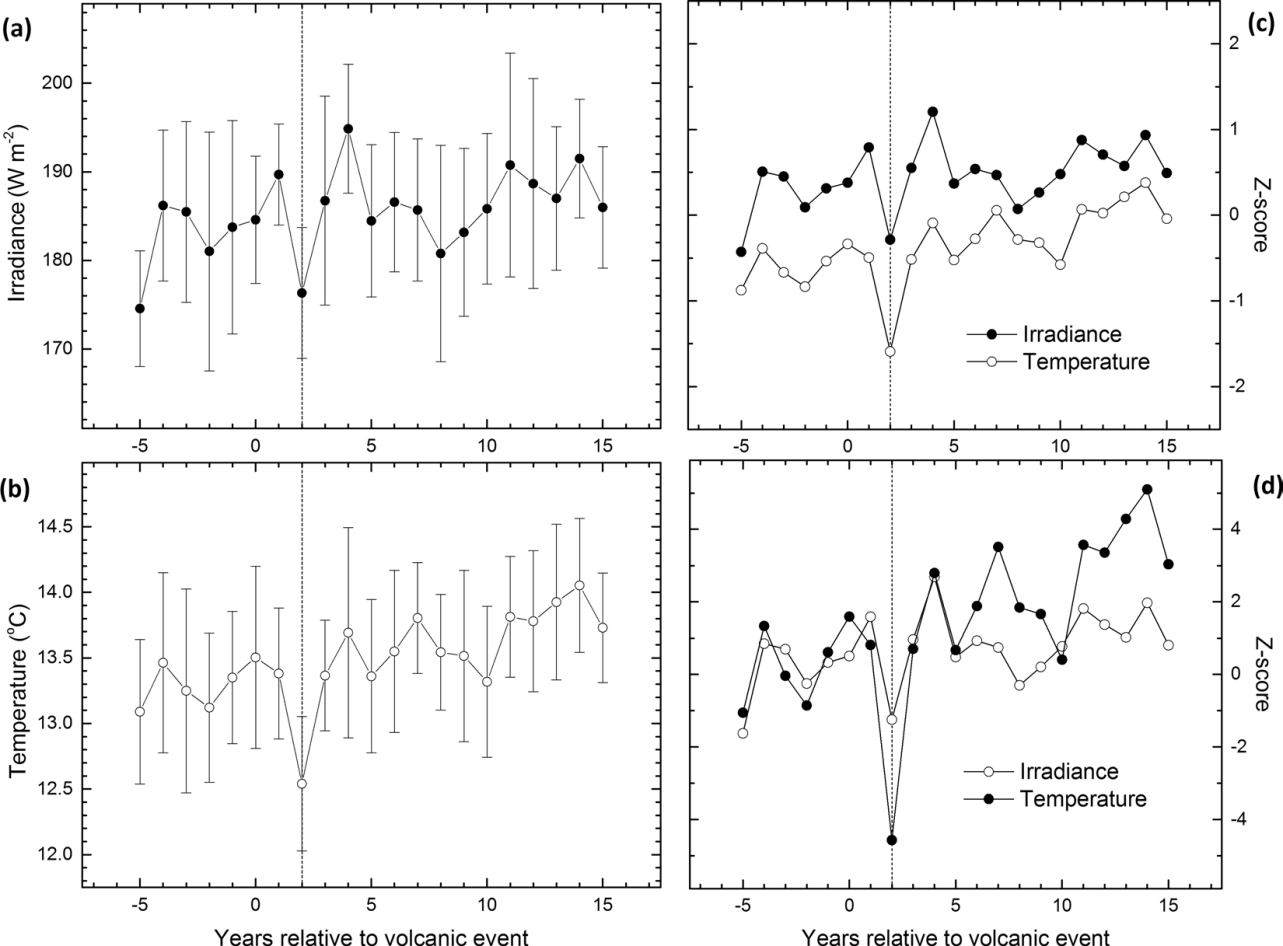
Superposed epoch analysis. Solar irradiance (this study) and northern Fennoscandian summer-temperature (June through August) reconstructions^37^ centered on eleven large volcanic eruptions of the past two centuries^34^ overlapping our living tree δ^13^C chronology (AD 1831–2011) expressed by their reconstructed values (**a**,**b**) and values normalized relative to the AD 1961–1990 period (**c**) and with respect to the five years preceding the volcanic events (**d**). Consistent with an expected response^34^, the strongest signal of temperature response during the summer season was demonstrated for year +2 (vertical dashed line). Our new chronology illustrates this response also as a reduction in solar irradiance.

Compared to the AD 536 event, the AD 541–544 drop in irradiation was substantially stronger, at least in absolute terms (Fig. 4A). These magnitudes were consistent with ice-core sulfur records with respect to the strength of global volcanic forcing and with the longer duration of sulfate deposition from the AD 540 eruption^2,13^. With regard to the pre-event (AD 519–535) values, the magnitudes of reconstructed irradiance change for AD 536 and AD 541–544 amount to 41 and 54 to 62 Wm^−2^ reductions, respectively. These losses represent values 2.5–3 standard deviations below the reconstructed overall mean values (Fig. S2). The strength of these anomalies is further demonstrated in that both the amplitude and duration of the AD 536 and AD 541–544 events respectively exceed the effects of eleven large eruptions^34^ experienced over the past two centuries by the living tree δ^13^C chronology (Figs 6; S3).

First-differencing the reconstruction data demonstrates the strength of sudden change in irradiance from AD 535 to 536 (Fig. 4B). That the change was apparently of lesser magnitude for AD 541 is understood in the sense of relatively low irradiance occurring already in AD 540. Other anomalies of similar magnitude occurred in AD 522, 527, 549 and 595. Three out of four of these irradiance events follow volcanic forcing from Northern Hemispheric or tropical eruptions in the same or previous year^2^. Irradiance anomaly of AD 527 exists with no connection to major eruption in ice-core sulfur record and may also represents a synoptic weather situation controlling the European irradiance anomalies^35^. Strong episodes of cooling are recorded in European^36^ or north Fennoscandian^37^ tree-ring proxies of maximum-latewood density chronologies in AD 536, 541 and 543–544 (Fig. 4C). The cooling in AD 574 follows the eruption dated to same year in ice-core record^2^ and was also accompanied by a notable reduction in irradiance (for other potential connections between volcanic forcing and irradiance anomalies, see Fig. S4).

## Discussion

Surviving writings describing the veiling of the solar radiation during and after AD 536 largely originates from the Mediterranean sources once created by court historians and chroniclers. In these writings the sun was observed blue-colored, without brightness, spring without mildness, summer without heat^10^. The overriding reason for these anomalies was the mystery cloud, a persistent dry fog that darkened the sky, the cloud that was observed by contemporaries over wide areas across the Palearctic all the way from the British Isles to China^11,38,39^. Palaeoclimate literature has for long attributed the cloud to volcanic aerosol emissions^6–8^. Moreover, the anomalous climates during the event have been largely described in terms of cold summers^8^ such conditions having probably lasted as a protracted, at least decadal event much over the Northern Hemisphere^12^. An essential point is that the existing palaeoclimatic inferences have thus far been extracted from tree-ring width/density chronologies that are proxies for past summer temperatures. However, the temperature effects remain subordinate to the primary diagnosis, the opaque skies and the vastly reduced sunlight under them^11^. As a consequence, the survey of the climate processes during the event has remained, at best, one-sided, and somewhat biased towards its temperature characteristics which, albeit playing an important role, may actually represent secondary effects. By contrast, the δ^13^C data we have presented facilitate the first quantification of sunlight conditions from year to year during the dust veil episode and make it possible to reconstruct the markedly varying solar radiation from subfossil tree rings. Importantly, our results provide several aspects of volcanic summers independent of temperature effects and contribute to our understanding of the event as a multifaceted climate crisis during which the adverse effects of cold temperatures may have been reinforced by strong reductions in irradiance with the hardship of rapid climate change on human societies.

Away from key regions of written evidence our results still demonstrate the dust veil to have resulted in low levels of irradiance not only in AD 536 but subsequent to the second (AD 540) (ref.^2^) eruption with reductions in irradiance nearly three standard deviations below the mean, and a continuation of low irradiance over the coming two decades. This evidence overlap with indications of cold summer temperatures persisting from AD 536 to 545 (ref.^12^), probably until the AD 570 s (ref.^14^), and suggests that the dust veil may have acted as a radiative forcing agent for the decadal cooling. We also note that previous literature has depicted coinciding δ^13^C anomalies in Siberian tree rings which may be an indication of extended spatial scale of the event, albeit the actual reasons behind the anomalies in Siberian data remain to be reconstructed^40^. Historical accounts of the dust veil concentrate on an 18-month period from March of AD 536 until June of AD 537 (refs^10^^,^^11^). The δ^13^C proxy data suggests an abrupt reduction in irradiance in AD 536, with even a more prolonged anomaly after the second eruption. We assume these patterns to reflect the potential spatiotemporal regimes in aerosol forcing through the anomalous AD 536–545 decade that are likely to differentiate the two eruption signals^2^. While the subarctic regions provide no documentation from that time the existing archaeological evidence from sites adjacent to ours do indicate crop failures, demographic catastrophe^41^, disruption of settlement, and population displacement^42^. Moreover, climate modelling demonstrates cold summer temperatures having decreased the agricultural production over the same areas, at least over the AD 536–545 decade^13^. In areas central to the mystery cloud observations, however, the plant products are less limited by temperature^19^. Clearly, some factor other than low temperature should have played a role in generating the severe damages to crop yields described in the written Mediterranean sources during the same years. Such damages are increasingly documented in the eastern Roman Empire around AD 536, recorded as the highest peak in the number of famines for that region and period, in the context of the Roman and post-Roman world from 100 B.C. until AD 800 (ref.^15^). In Mediterranean-type environments, the sum of solar radiation increases the growing season length and the production of crop dry matter depends primarily on the amount of solar radiation^43^. Our results add to this stream of research by quantifying low irradiance during the years topical to the human consequences thus providing regulatory mechanism to crop reductions with direct links from volcanic dust veil to food crises over wide geographical scales.

Yet another factor possibly causing the crop failures and famines was the drought described in the written sources, according to which the winter (conceivably that of AD 536/537), or possibly later part of the year, was rendered dry and without storms^10^. Our δ^13^C data remain sensitive to summer climate^27^ and cannot comment on any wintertime anomalies. Moreover, the coming of drought would not be consistent with hypothetical post-eruption hydroclimate summertime responses, expected to mimic the configuration of North Atlantic pressure fields during the negative phase of the East Atlantic (EA) teleconnection pattern^44^, and to result in wet conditions around the Mediterranean realm^44–46^. The EA pattern is the leading mode of climate variability over the North Atlantic and surrounding continents representing a north-south dipole of pressure centers^47^. The EA is negatively correlated with instrumental precipitation/soil moisture across the Mediterranean, the respective correlations in the region of our δ^13^C evidence remaining slightly positive but non-significant during the summer months^44,48^ (see Fig. S5 for correlations with could cover). Assuming that the mid-sixth century eruptions were followed by similar, negative EA phase (there are currently no palaeoclimate EA reconstructions available for the first millennium), the presented correlations^44,48^ would agree with our palaeoecological model, suggesting a negligible hydroclimatic response over the tree-ring sites during the event years.

The years of strongest sunlight deficiency coincides remarkably with the years of Justinianic plague (AD 541–544), the first historically recorded outbreak of true plague that ravaged the Mediterranean world^9,49,50^. After the first epidemic that broke out in Constantinople, the eastern Roman Empire, the pandemic spread widely to the entire Mediterranean and central Europe, as north as Finland^51^, killing tens of millions until the mid-8th century^52^. Volcanic forcing triggering the climatic cooling arguably provoked the first impulse to the plague in AD 541 (ref.^9^). The plague was possibly introduced to Constantinople by rats and fleas infected by *Yersinia pestis* bacteria travelling on board from Egypt. Moreover, it is hypothesised that the exceptionally cool weather of AD 541 was beneficial to rat survival and flea reproduction. As a consequence, the climatic event appears having played a role in creating an unusual opportunity for animal vectors to cross the Mediterranean^52^. The recent historical evidence for coinciding high number of famines over the same region suggests that the nutritional background may have contributed to the explosion of the plague^15^. The interval of strongest sunlight deficiency in our reconstruction, AD 541–544, coincides strikingly with the years of the Justinianic plague and help to explain the environmental drivers of the chronic food shortages. In addition to undernutrition, we hypothesize that the death toll may have been raised by adverse effects from deficiency symptoms of vitamin D. Photosynthesis of the vitamin D in human skin occurs only when a certain threshold of incident solar radiation is exceeded and can halt completely even at the equator under a very thick overcast cloud^53^. Topically, adequate vitamin D status is important for overall health and well-being with positive effects on the immune system, for example, in the case of bacterial infections^54^. Atmospheric aerosols attenuate incoming solar ultraviolet radiation at the Earth’s surface^53^ and the volcanic dust veil such as that indicated from our reconstruction between AD 541 and 544 could thus have contributed to human consequences by environmentally modifying the chemical reactions directly in humans. Although no climatic proxy may be adequate to validate such effects on vitamin status, it seems plausible that the photosynthesis of the vitamin D may have been reduced in human skin during the irradiance anomaly. Translating into suboptimal vitamin D status, the low levels of irradiance under the volcanic dust veil may have hypothetically predisposed the contemporaries to bacterial infection by *Yersinia pestis* at the same time the cold climates assisted the bacteria to land the Roman Empire.

Collectively, our data confirm abrupt changes to the growth seasons (i.e., summers) following the large volcanic eruptions in AD 536 and 541–544 in the form of cooling and, more importantly, strong reduction in incoming solar radiation. During and after these events, the cooling was likely driven by the dust veil and photosynthetic products were limited to such an extent that they likely affected food security and human immune system. These findings add to our knowledge of volcanic aerosol forcing and emphasize their importance with respect to the temperature-related scenarios frequently described in the literature. Understanding the multifaceted environmental impacts of ancient explosive eruptions requires the use of proxy data that are sensitive to variable irradiance levels and which faithfully track this vital component of a productive ecosystem. Our results underscore the pressing need for a database of tree-ring isotope chronologies and archaeological/historical records in order to investigate the relationship between irradiation and human society. Only these data can describe the spatial and temporal variation in volcanic aerosol emission forcing from one event to another and allow them to be compared to accounts from the regional to continental scale. Nevertheless, we endorse the need of combining the data of climate forcings with thorough analyses of political and economic structures^15,16,49,50^ of which determinants have almost certainly contributed to the event. Such a comparison would reveal the relative impact(s) of climatic forcing on agriculture, human health, urbanisation and movement during the first millennium – a period considered to contain the main ‘hinges’ of human history^55^.

## Methods

Our sampling area is situated in the Finnish Lapland in northern Europe. Tree-ring dated samples (see Fig. S6) were dissected from subfossil and living Scots pine (*Pinus sylvestris* L.) collected from the timberline and forest protection areas of Lapland^56^. Cross-dating of our tree-ring series against the existing mean chronology^57^ enables the dating of each ring to the exact calendar years. Tree-ring samples were processed to α-cellulose using two alkaline extractions (5–7% NaOH) and a chlorination step (NaClO_2_) in between^58^, homogenized using an ultrasonic probe^59^, and freeze-dried. These dry cellulose fibres (ca. 70 μg) were combusted for isotopic analysis on a DeltaPlusAdvantage isotope ratio mass spectrometer coupled to a CN2500 elemental analyzer at the Laboratory of Chronology, University of Helsinki. All samples were analyzed in duplicate, and randomly selected samples were subsampled for 10 replicate analyses to monitor efficiency of homogenization and result reproducibility. The δ-notation as per mille (‰) expresses the deviations from the VPDB standard. The mean reproducibility of analyses was ±0.1‰, estimated from sample replicates and 75 repeated analyses of an internal laboratory reference cellulose (Fluka-22181 cellulose powder, Sigma-Aldrich, Lot. 442654/1) analyzed alongside sample material. The raw δ^13^C data is made available at the National Centers for Environmental Information – National Oceanic and Atmospheric Administration (https://www.ncdc.noaa.gov/data-access/paleoclimatology-data). The δ^13^C data were corrected for changes in δ^13^C value of atmospheric CO_2_ due to the industrial revolution^60^ and discrimination rate changes by 0.0073‰ per ppmv CO_2_ (ref.^61^); the reliability and validity of which have been established^27^ and found to be consistent with these two independent methods of correction^62,63^. Trends related to tree age rather than climate variability were previously documented for δ^13^C data^33^ and removed here using the regional curve standardization^64^ known to preserve the full spectrum of short-to-long timescale climate information. Each δ^13^C value was standardized by subtracting age-dependent δ^13^C value from the δ^13^C value and by adding a constant (−24.9‰) representing the overall mean value of the δ^13^C data. Arithmetic mean was used to build the mean δ^13^C chronology.

The results were interpreted in keeping with the Farquhar model of photosynthesis^22,23^ describing the δ^13^C in wood material as

1 $${\delta }^{13}C={\delta }^{13}{C}_{ATM}-a-(b-a){c}_{i}/{c}_{a}$$

where *δ*^13^*C*_*ATM*_ represents the δ^13^C value of CO_2_ in the ambient air, *a* the diffusional fractionation, *b* carboxylation fractionation, and *c*_*i*_*/c*_*a*_ the ratio between the concentration of CO_2_ in intercellular spaces (*c*_*i*_) and in the air (*c*_*a*_). According to the model, more negative δ^13^C values are expected when factors increasing *c*_*i*_*/c*_*a*_ such as low light intensity lowering the rate of photosynthesis lead to a rise in *c*_*i*_. Such conditions may be expected after explosive volcanic eruptions as their aerosol emissions reduce solar radiation, as observed in our subfossil δ^13^C data in AD 536 and 541–544. Less negative δ^13^C values are expected when drier conditions lead to a higher degree of stomatal closure and hence, according to the model, decreased *c*_*i*_. Consistent with this premise, the subfossil δ^13^C values of the upland site are less negative than those of the riparian sites and thus reflect the drier soil conditions at upland sites. We note that the riparian and upland δ^13^C data correlate positively (Fig. S1) and see no verifiable reason the riparian and upland pines had reacted disproportionately to factors other than moisture. Thus, the moisture signal present in the upland δ^13^C series may be statistically extracted from the riparian δ^13^C data using a regression model. Entering upland δ^13^C as independent and riparian δ^13^C as dependent data, we obtain a new δ^13^C series (δ^13^C_NEW_) in the form of residuals from the model as

2 $${\delta }^{13}{C}_{NEW}={\delta }^{13}{C}_{RIPARIAN}-(0.379\times {\delta }^{13}{C}_{UPLAND}-15.810)-24.9\textperthousand $$

where the parameters of 0.379 and −15.810 are the slope and intercept obtained respectively from fitting a linear regression model to the data and −24.9‰ represents the overall mean value of the data. We observed the anomalies in AD 536 and 541–544 also in the new δ^13^C data as evidence to suggest that this signal is unrelated to moisture fluctuations and represents a dry fog event akin to a volcanic dust veil.

Living pines were sampled from the same sites as subfossil so as to maximize the reciprocal comparability with subfossil samples. Thus, the sampled trees represent the sites from Näkkälä, Kultima, Luolajärvi, and Autsasenkursu^27^. The annual δ^13^C modern chronology was calibrated against the measured sunlight data (global radiation in June–July season^27^) as observed at the local meteorological station^65^, Sodankylä, Finland; 67.37°N, 26.65°E, WMO station code: 2836) (https://en.ilmatieteenlaitos.fi/open-data/) and using a linear regression over a 40-year common period (AD 1971–2011). We note that this variable (i.e., global radiation) refers to the total solar radiation received from the sky on a horizontal surface and not to the global spatial scale. Combined with visual comparisons of reconstructed and observed series (Fig. 3), the calibration and verification statistics applied separately over the early (AD 1971–1991) and late (AD 1992–2011) periods were found to successfully evaluate the reliability of the reconstruction (Table S2). The applied statistics included the coefficient of determination, reduction of error, and coefficient of efficiency^66^. We follow the established Monte Carlo framework^67^ of testing the statistical significance of these variables using the published algorithms^68^ downloadable at http://www.helsinki.fi/science/dendro/reconstats.html. The final reconstruction model (i.e., the transfer function built over the AD 1971–2011 period) explained more than half of the variance in the instrumental data (R^2^ = 0.522, p < 0.0001).Thus, our δ^13^C dataset exceeded the thresholds previously set for an acceptable δ^13^C proxy^28,29^ and jack-knifed estimates of the δ^13^C chronology confirmed the robustness of the calibration (Table S2). We also tested the δ^13^C chronology against the record of diffuse radiation over the same June–July season as above, observed at the same weather station (Sodankylä). These data are available through the World Radiation Data Centre database (http://wrdc.mgo.rssi.ru/) and cover 1991–2011 ad. With these regards, our δ^13^C data correlate highly with global radiation but show virtually no association with the diffuse radiation data (Fig. 5). The final model was parameterized as

3 $${I}_{t}=35.7\times {\delta }^{13}{C}_{t}+1059.5$$

where the annual summer irradiance (*I*) was reconstructed from the proxy data of mean δ^13^C chronology in the same year (*t*). Applying the transfer function for the subfossil δ^13^C data provides an estimate of palaeo-sunlight variability during the large volcanic eruptions of the AD mid-sixth century (Fig. 2). The series of reconstructed irradiance values and their confidence intervals are provided in Table S3. Summer irradiance is limited by variations in cloud cover and our reconstruction correlates with r = −0.505 (p < 0.0001) with cloud cover variability in the same June–July season as observed at the Sodankylä meteorological station over the AD 1908–2011 period. There is a very similar association found even over earlier period of cloud cover observations made in the region in Matarenki–Övertorneå (66.38° N, 23.67° N) from October AD 1802 until December AD 1838 (ref.^69^). Averaged over the June–July season this cloud cover record correlates with our reconstruction with r = −0.523 over their common period (AD 1831–1838).

Superposed epoch analysis was used to assess the climate anomalies characteristic of the post-eruption sequence (Fig. 6) as commonly applied to studies of temperature change following historical volcanic eruptions^34^. Solar radiation (this study) and north Fennoscandian summer (June–August (JJA)) temperature, based on the maximum-latewood density (MXD) chronologies^37^, were centered on 11 large volcanic eruptions of the past 200 years^34^. These eruptions had volcanic explosive index^70^ values of five or more and thus likely associated with climatic effects at the hemispheric scale as previously shown for summer cooling^34^. In order to demonstrate that the value of δ^13^C and MXD chronologies to reconstruct past variations in irradiance and temperature, respectively, the two types of tree-ring chronologies were correlated against the JJA irradiance and mean average temperature records obtained from the same meteorological station (Sodankylä) as used in the main analyses. To do so, the JJA temperature-dependence was regressed out from the JJA irradiance record as residuals from the linear regression model, such residuals representing temperature-independent JJA irradiance. Likewise, the influence of JJA irradiance was regressed out from the JJA temperature. The new, residual irradiance series correlated statistically significantly only with δ^13^C data, whereas the residual temperature series correlated statistically significantly only with MXD data (Fig. S7). Previously, the mean maximum temperature was found to more strongly relate to the δ^13^C data than mean average temperature^27^. The analyses were repeated using this variable and found to yield highly similar results (Fig. S8). These relationships demonstrated the value of our δ^13^C and MXD chronologies as proxies for summer irradiance and temperature, respectively.

## Electronic supplementary material

Supplementary Information
