# Supplementary material for: Volcanic dust veils from sixth century tree-ring isotopes linked to reduced irradiance, primary production and human health
Source: Sci Rep. 2018 Jan 22;8:1339. doi: 10.1038/s41598-018-19760-w (PMC5778132; doi:10.1038/s41598-018-19760-w)
Supplement: Supplementary file 1 — Supplementary Information [file 41598_2018_19760_MOESM1_ESM.pdf]

Volcanic dust veils from sixth century tree-ring isotopes linked to reduced irradiance, primary production and human health

Samuli Helama, Laura Arppe, Joonas Uusitalo, Jari Holopainen, Hanna M. Mäkelä, Harri Mäkinen, Kari Mielikäinen, Pekka Nöjd, Raimo Sutinen, Jussi-Pekka Taavitsainen, Mauri Timonen & Markku Oinonen

Supplementary Information

Content

- Figure S1: Correlations between the $\delta^{13}C$ series
- Figure S2: Irradiance reconstruction
- Figure S3: Superposed epoch analysis
- Figure S4: Reductions in irradiance and volcanic events
- Figure S5: Cloud cover and the East Atlantic pattern
- Figure S6: Tree-ring dating
- Figure S7: Irradiance and mean average temperature
- Figure S8: Irradiance and mean maximum temperature

- Table S1: Site characteristics
- Table S2: Calibration and verification statistics
- Table S3: Reconstructed irradiance

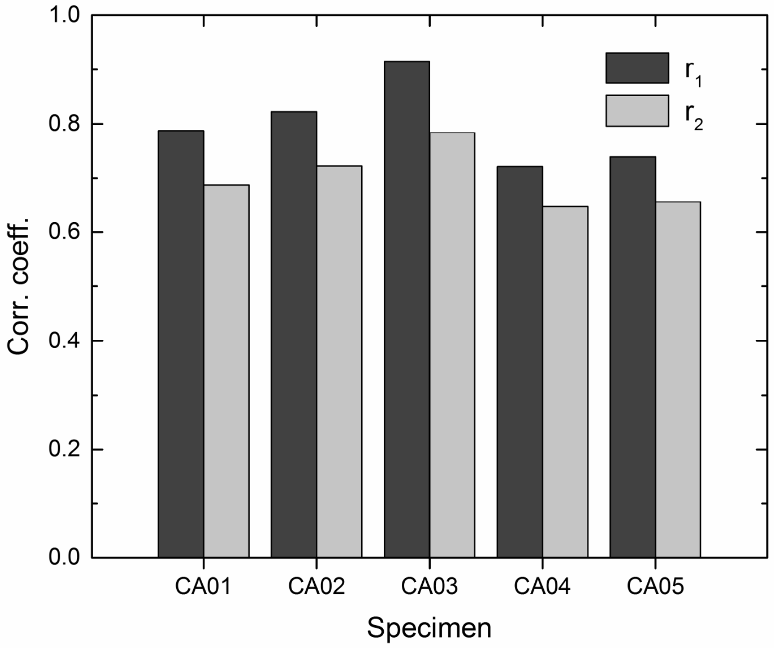

Figure S1. Correlations between the subfossil $\delta^{13}C$ series. Linear (Pearson) correlations among the subfossil specimens illustrate their signal strength over the timeframe (AD 532–551) of volcanic events. Pearson correlations were assessed between each of the series to the mean of the other $\delta^{13}C$ series (r_1) and as mean inter-series correlations (r_2).

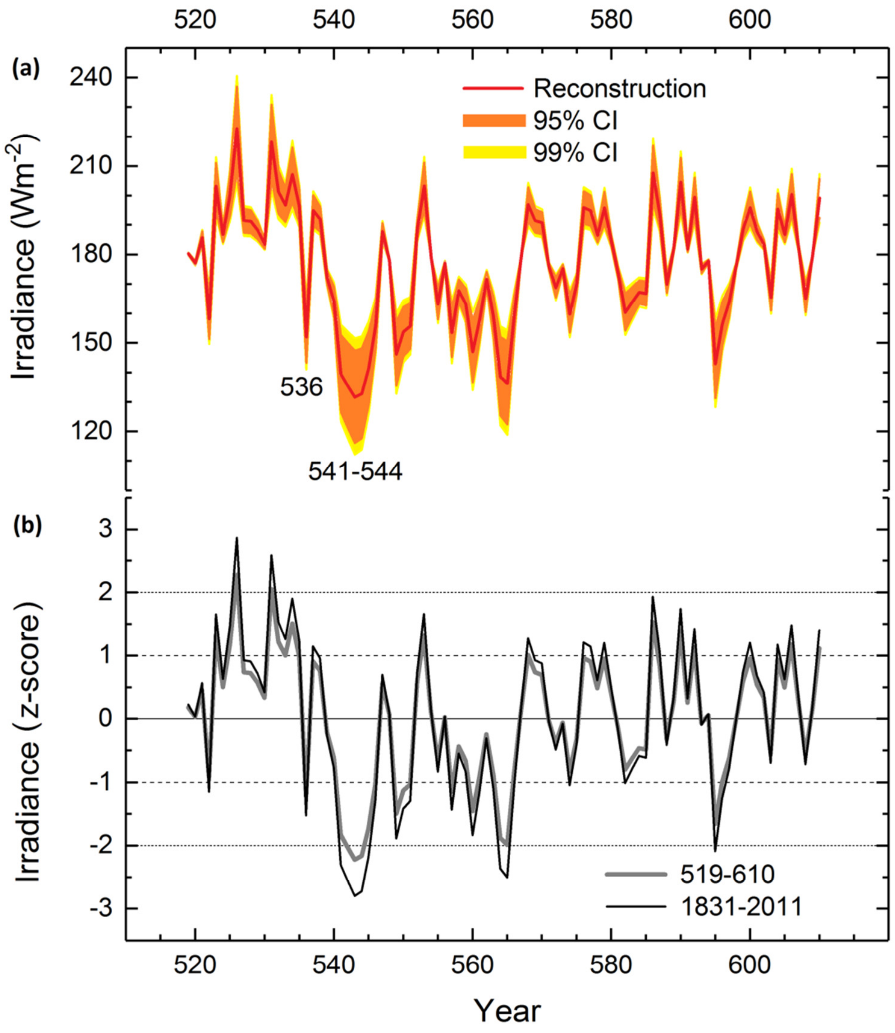

Figure S2. Irradiance reconstruction. The new tree-ring $\delta^{13}C$ based reconstruction of solar irradiance (global radiation) (red line) with Monte Carlo based estimates of 95% (orange area) and 99% confidence (yellow area) intervals (a) transformed into z-scores, with a mean of zero and standard deviation of one, relative to the living tree (AD 1831–2011) and subfossil (AD 519–610) baseline values (b).

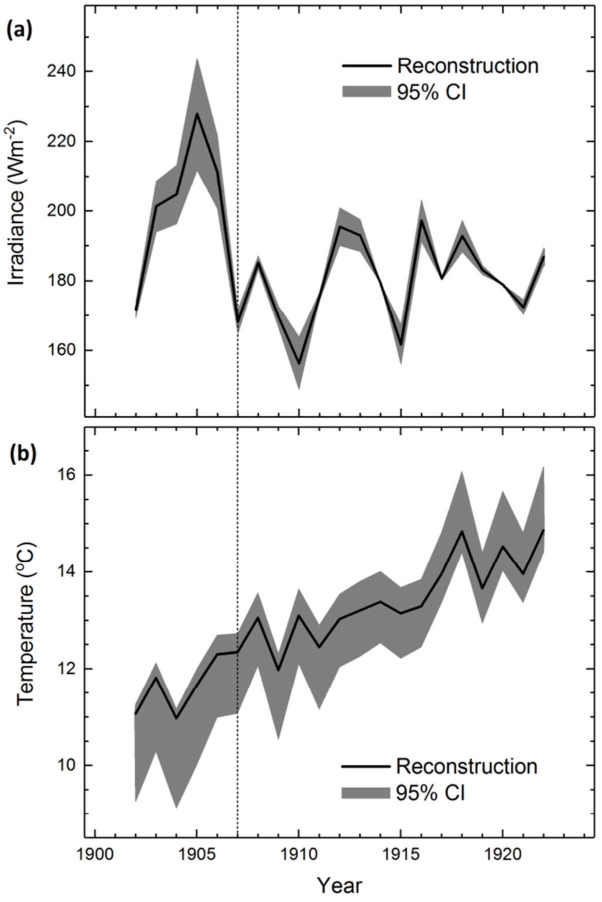

Figure S3. Reconstructed post-eruption change. Tree-ring based solar irradiance (this study) (a) and northern Fennoscandian summer-temperature (June through August) reconstructions[37] (b), with estimates of 95% (gray area) confidence intervals, relative to Ksudach (Russia) eruption in March AD 1907 (vertical dashed line), representing the strongest post-eruptive reduction in reconstructed irradiance data over the living tree (AD 1831–2011) period. In contrast, the response in summer temperature appears largely masked by the overlapping trend of early 20th century warming.

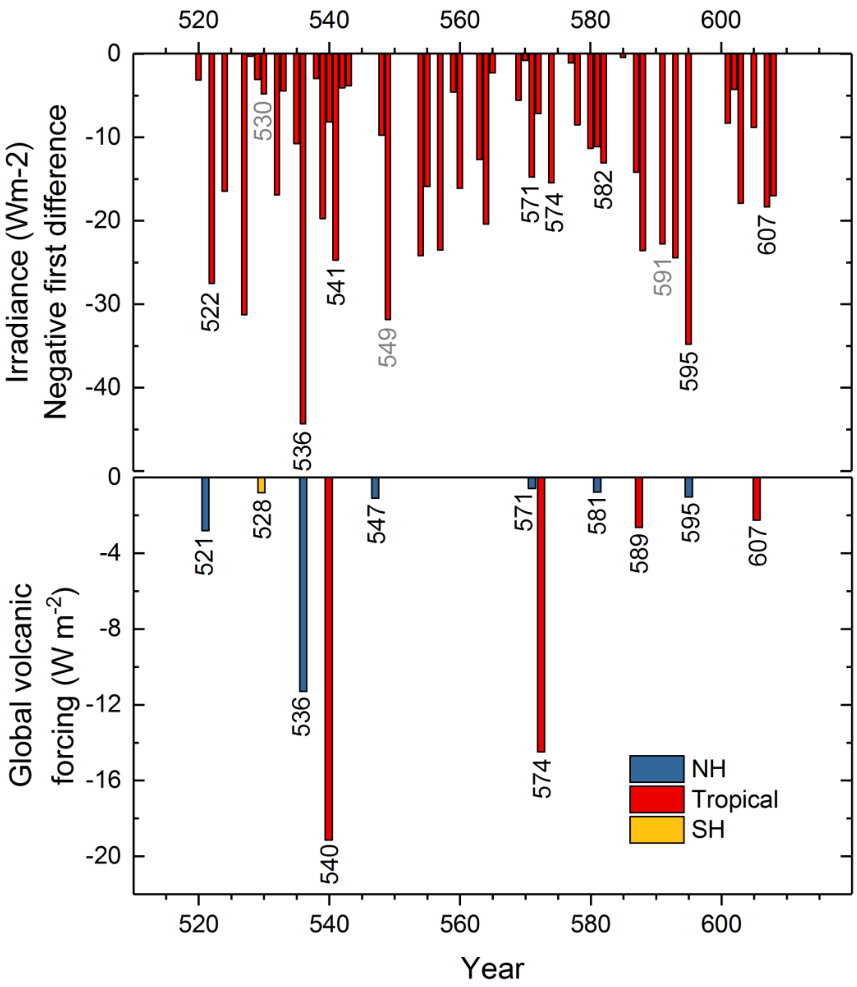

Figure S4. Reductions in irradiance and volcanic events. Negative first difference of the reconstructed irradiance recording the change in irradiance from previous to concurrent year (this study) compared with the volcanic aerosol forcing events from ice core evidence with list of eruptions of Northern Hemisphere (NH), tropical and Southern Hemisphere (SH) origin over sixth century[2]. The years with strongest post-eruptive irradiance anomaly with lag of 0–1 year indicated with black ink, the years with lag of 2 yeas indicated with gray ink.

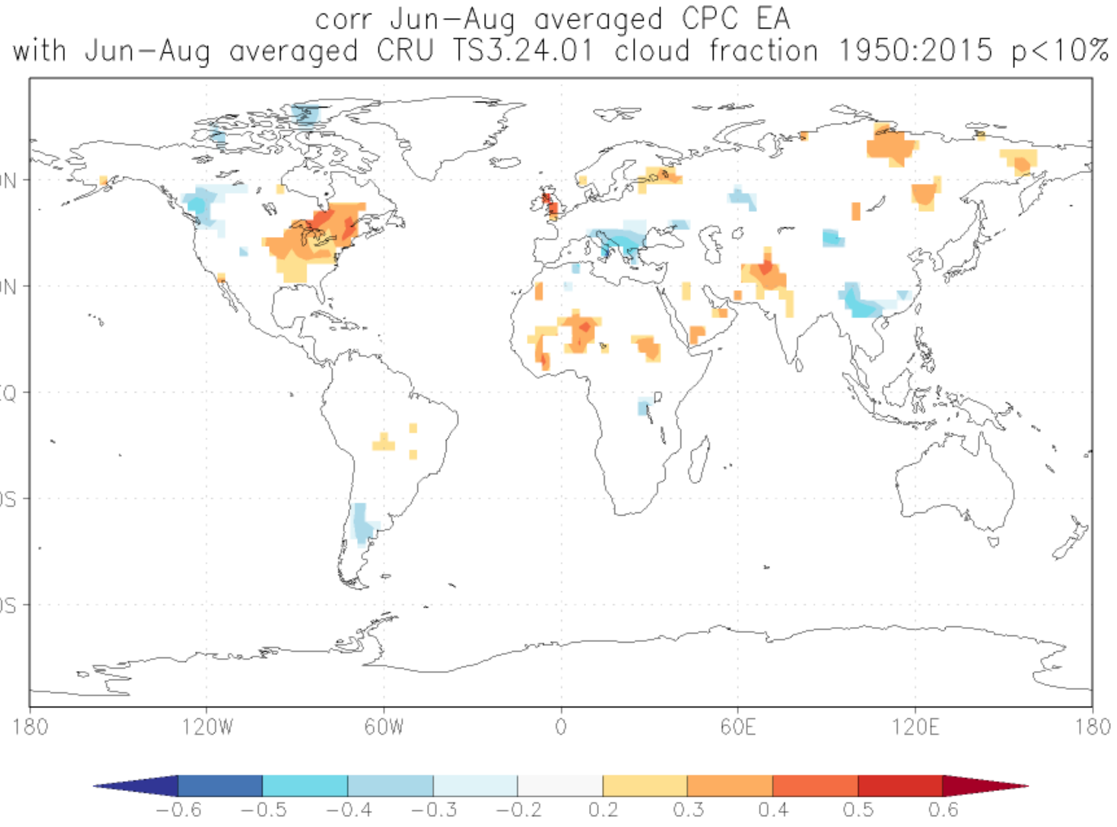

Figure S5. Spatial correlations. Correlations were calculated between the summertime cloud cover (Harris, I. *et al. Int. J. Climatol.* 34**,** 623–642 (2014)) and East Atlantic pattern[47] produced at the https://climexp.knmi.nl/ (Trouet, V. & van Oldenborgh, G. J. *Tree-Ring Res.* 69**,** 623–642 (2013)).

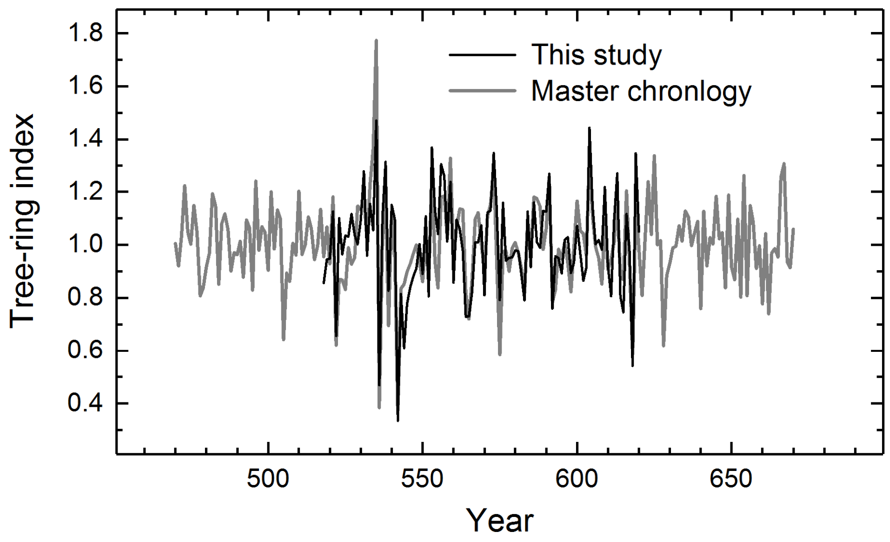

Figure S6. Tree-ring dating. Comparison between the mean chronology of our *Pinus sylvestris* samples and the existing master chronology[57]. The ring-width data were converted into tree-ring indices using the dating algorithms (Holmes, R. L. *Tree-Ring Bull.* 43**,** 69–78 (1983)).

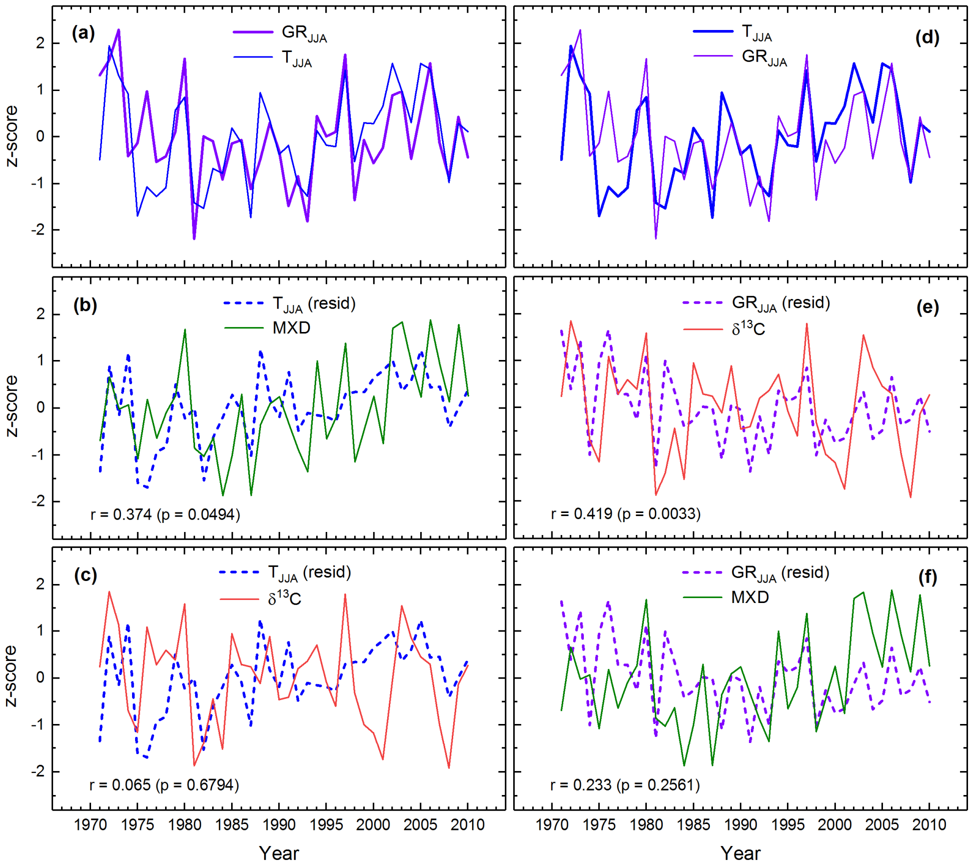

Figure S7. Irradiance and mean average temperature. Mean average summer (June–August) temperature (T_{JJA}) data was regressed against the global radiation (GR_{JJA}) over same season (r = 0.642) (a), and the residuals from that model (T_{JJA} (resid)) correlated with the maximum-latewood density (MXD) chronology[37] (b) and with the $\delta^{13}C$ chronology of this study (c). The GR_{JJA} data was regressed against the T_{JJA} data (d), and the residuals from that model (GR_{JJA} (resid)) correlated with the $\delta^{13}C$ (e) and the MXD records (f). Statistical significance (p) of Pearson correlations (r) was assessed using the Monte Carlo simulations[68].

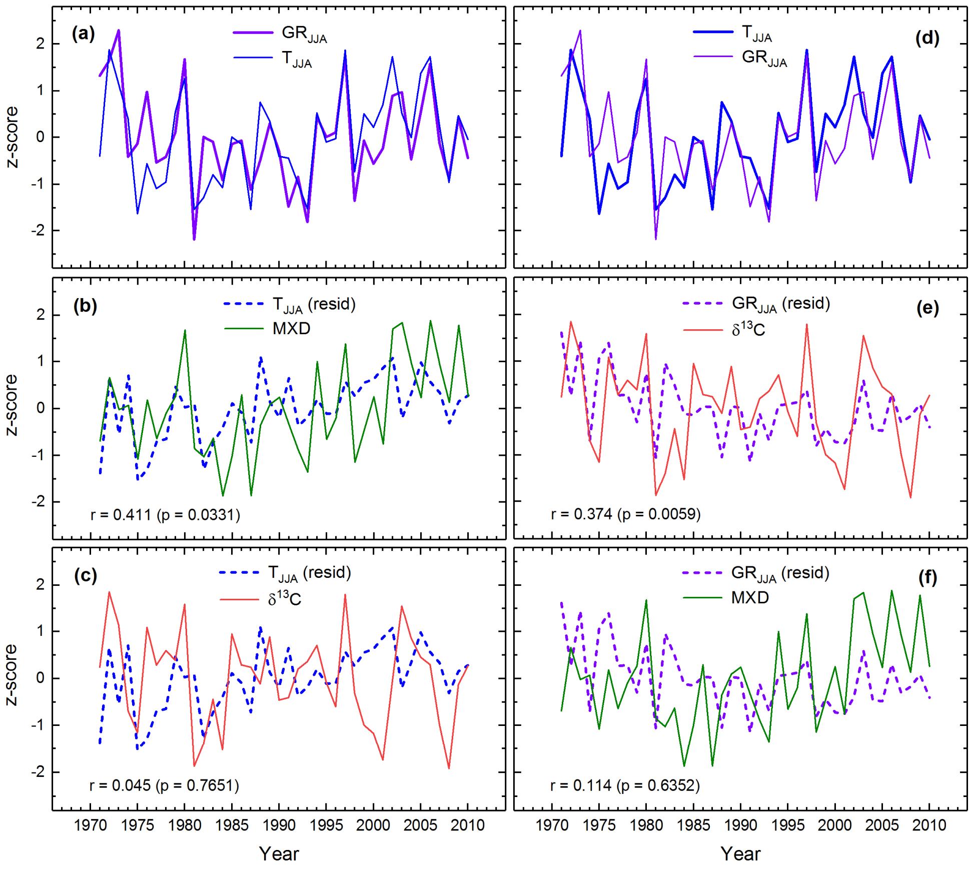

Figure S8. Irradiance and mean maximum temperature. Mean maximum summer (June–August) temperature (T_{JJA}) data was regressed against the global radiation (GR_{JJA}) over same season (r = 0.737) (a), and the residuals from that model (T_{JJA} (resid)) correlated with the maximum-latewood density (MXD) chronology[37] (b) and with the $\delta^{13}C$ chronology of this study (c). The GR_{JJA} data was regressed against the T_{JJA} data (d), and the residuals from that model (GR_{JJA} (resid)) correlated with the $\delta^{13}C$ (e) and the MXD records (f). Statistical significance (p) of Pearson correlations (r) was assessed using the Monte Carlo simulations[68].

Table S1. Site characteristics.

Specimen	Lat	Long	Alt	Site	Habitat	Position
CA01	68.57	23.48	366	Näkkälä	Riparian	Timberline
CA02	68.47	22.96	343	Kultima	Riparian	Forest
CA03	68.47	28.00	183	Luolajärvi	Riparian	Forest
CA04	68.55	22.14	382	Autsasenkursu	Upland	Timberline
CA05	68.57	23.48	366	Näkkälä	Riparian	Timberline

Sites are described for their latitude, longitude, altitude (Alt; meters above sea level), habitat and position.

Table S2. Calibration and verification statistics.

Statistic	Calibration	Verification	$\delta^{13}C_{FULL}$	$\delta^{13}C_{JK-01}$	$\delta^{13}C_{JK-02}$	$\delta^{13}C_{JK-03}$	$\delta^{13}C_{JK-04}$	$\delta^{13}C_{JK-05}$
R^2	1971–2011		0.522	0.528	0.519	0.432	0.474	0.542
p	1971–2011		<0.0001	<0.0001	0.0001	<0.0001	<0.0001	<0.0001
R^2	1992–2011	1971–1991	0.536	0.535	0.548	0.439	0.508	0.558
p	1992–2011	1971–1991	0.0014	0.0011	0.0013	0.0065	0.0008	0.0006
r^2	1992–2011	1971–1991	0.517	0.547	0.498	0.445	0.439	0.531
p	1992–2011	1971–1991	0.0017	0.0019	0.0026	0.0049	0.0061	0.0018
RE	1992–2011	1971–1991	0.480	0.483	0.431	0.394	0.387	0.501
p	1992–2011	1971–1991	0.0004	0.0010	0.0007	0.0014	0.0012	0.0001
CE	1992–2011	1971–1991	0.474	0.477	0.424	0.387	0.379	0.495
p	1992–2011	1971–1991	0.0002	0.0007	0.0005	0.0008	0.0005	0.0001
R^2	1971–1991	1992–2011	0.517	0.547	0.498	0.445	0.439	0.531
p	1971–1991	1992–2011	0.0017	0.0019	0.0026	0.0049	0.0061	0.0018
r^2	1971–1991	1992–2011	0.536	0.535	0.548	0.439	0.508	0.558
p	1971–1991	1992–2011	0.0014	0.0011	0.0013	0.0065	0.0008	0.0006
RE	1971–1991	1992–2011	0.512	0.501	0.514	0.411	0.477	0.537
p	1971–1991	1992–2011	0.0003	0.0004	0.0003	0.0003	0.0002	0.0001
CE	1971–1991	1992–2011	0.509	0.498	0.510	0.407	0.474	0.533
p	1971–1991	1992–2011	0.0002	0.0002	0.0002	0.0001	0.0001	0.0001

Reconstructions of global radiation were derived from the chronology with full set of $\delta^{13}C$ data ($\delta^{13}C_{FULL}$) and from the jack-knifed ($\delta^{13}C_{JK}$) estimates of the $\delta^{13}C$ chronology. Calibrations are quantified using the coefficient of determination (R^2) and verifications using the squared correlation coefficient (r^2), the reduction of error (RE) and the coefficient of efficiency (CE) statistics with their obtained Monte Carlo based[68] statistical significance (p).

Table S3. Reconstructed irradiance (Wm^{-2}) values with their Monte Carlo based estimates of confidence intervals (CI).

Year	Irradiance	Lower 95% CI	Upper 95% CI	Lower 99% CI	Upper 99% CI
519	180.2	179.8	180.6	179.7	180.7
520	177.1	176.4	177.8	176.3	177.9
521	185.6	183.5	187.8	182.9	188.3
522	158.1	151.3	165.1	149.6	167.0
523	203.2	195.2	211.0	193.0	213.0
524	186.7	184.2	189.2	183.5	189.8
525	200.2	193.2	207.0	191.3	208.7
526	222.7	208.2	236.9	204.3	240.5
527	191.5	187.3	195.5	186.3	196.5
528	191.2	187.2	195.1	186.1	196.1
529	188.1	185.1	191.0	184.3	191.8
530	183.3	181.9	184.7	181.5	185.0
531	218.1	205.2	230.8	201.7	234.1
532	201.3	193.9	208.5	191.9	210.3
533	196.8	190.9	202.6	189.4	204.1
534	207.1	197.8	216.3	195.3	218.6
535	196.4	190.6	202.0	189.1	203.4
536	152.0	143.2	161.0	141.0	163.4
537	195.0	189.7	200.1	188.3	201.4
538	192.0	187.7	196.2	186.6	197.3
539	172.3	170.1	174.5	169.5	175.2
540	164.1	159.3	169.1	158.0	170.4
541	139.4	126.5	152.6	123.2	156.1
542	135.3	121.1	149.9	117.5	153.8
543	131.5	116.1	147.4	112.1	151.6
544	132.7	117.6	148.1	113.8	152.3
545	141.4	129.2	154.0	126.1	157.3
546	156.0	148.5	163.7	146.6	165.7
547	187.7	184.9	190.5	184.1	191.3
548	178.0	177.6	178.4	177.6	178.5
549	146.2	135.5	157.1	132.7	160.0
550	153.7	145.5	162.2	143.4	164.4
551	155.7	148.0	163.5	146.1	165.5
552	188.4	185.3	191.5	184.5	192.2
553	203.3	195.2	211.1	193.1	213.1
554	179.1	179.0	179.1	179.0	179.1
555	163.2	158.0	168.5	156.7	169.9
556	177.0	176.3	177.7	176.1	177.9
557	153.5	145.2	162.0	143.1	164.3
558	167.7	164.0	171.5	163.1	172.5
559	163.1	157.9	168.4	156.6	169.9
560	147.0	136.6	157.7	134.0	160.5
561	158.0	151.1	165.0	149.4	166.9
562	171.6	169.1	174.1	168.5	174.8

563	158.9	152.4	165.6	150.7	167.4
564	138.5	125.3	152.0	122.0	155.6
565	136.3	122.3	150.5	118.8	154.3
566	159.5	153.2	166.0	151.5	167.8
567	179.4	179.3	179.4	179.2	179.5
568	197.0	191.0	202.8	189.5	204.3
569	191.5	187.3	195.5	186.2	196.5
570	190.6	186.8	194.4	185.8	195.4
571	175.9	174.8	176.9	174.6	177.2
572	168.7	165.3	172.2	164.5	173.1
573	175.2	174.0	176.5	173.7	176.9
574	159.8	153.5	166.2	151.9	167.9
575	170.0	167.1	173.0	166.3	173.8
576	196.0	190.4	201.5	188.9	202.9
577	194.9	189.6	200.1	188.2	201.4
578	186.4	184.0	188.8	183.3	189.4
579	195.8	190.3	201.3	188.8	202.7
580	184.5	182.7	186.3	182.2	186.7
581	173.4	171.5	175.3	171.1	175.8
582	160.3	154.2	166.6	152.7	168.2
583	163.8	158.9	168.9	157.6	170.3
584	167.1	163.3	171.1	162.3	172.2
585	166.7	162.7	170.8	161.7	171.9
586	207.6	198.1	216.9	195.6	219.3
587	193.5	188.7	198.1	187.4	199.3
588	169.9	166.9	173.0	166.2	173.8
589	182.0	181.0	182.9	180.8	183.2
590	204.5	196.1	212.8	193.8	214.9
591	181.8	180.9	182.6	180.6	182.8
592	199.4	192.7	206.1	190.9	207.7
593	175.0	173.7	176.4	173.3	176.7
594	177.8	177.3	178.2	177.2	178.3
595	143.0	131.2	155.0	128.2	158.2
596	156.5	149.1	164.0	147.2	166.0
597	163.9	159.0	169.0	157.7	170.3
598	176.2	175.2	177.2	175.0	177.4
599	188.5	185.4	191.6	184.6	192.4
600	195.9	190.3	201.3	188.8	202.7
601	187.6	184.8	190.3	184.0	191.0
602	183.3	181.9	184.7	181.5	185.0
603	165.4	161.0	170.0	159.9	171.2
604	195.4	190.0	200.7	188.5	202.1
605	186.6	184.1	189.1	183.5	189.7
606	200.4	193.3	207.3	191.4	209.1
607	182.0	181.1	183.0	180.8	183.3
608	165.0	160.5	169.7	159.3	171.0
609	179.6	179.4	179.7	179.4	179.8
610	199.1	192.5	205.7	190.7	207.3

1831	208.4	198.7	218.1	196.0	220.7
1832	188.5	185.4	191.6	184.5	192.4
1833	153.9	145.6	162.2	143.3	164.5
1834	154.8	146.9	162.9	144.7	165.1
1835	178.7	178.5	178.8	178.5	178.8
1836	187.7	184.8	190.5	184.1	191.3
1837	176.8	176.0	177.5	175.8	177.8
1838	198.1	191.8	204.4	190.1	206.1
1839	203.6	195.5	211.6	193.2	213.8
1840	213.5	202.1	224.8	199.0	227.9
1841	206.0	197.1	214.9	194.6	217.3
1842	191.2	187.2	195.2	186.1	196.3
1843	176.3	175.4	177.3	175.2	177.5
1844	205.6	196.8	214.3	194.4	216.6
1845	215.1	203.2	227.0	199.9	230.2
1846	186.8	184.3	189.4	183.6	190.1
1847	202.7	194.9	210.5	192.8	212.6
1848	167.3	163.5	171.2	162.4	172.3
1849	180.1	179.8	180.4	179.7	180.5
1850	188.8	185.6	192.0	184.7	192.8
1851	169.5	166.4	172.7	165.5	173.6
1852	192.8	188.3	197.4	187.0	198.6
1853	172.6	170.4	174.7	169.8	175.3
1854	190.9	187.0	194.7	185.9	195.8
1855	183.5	182.1	185.0	181.7	185.4
1856	167.5	163.7	171.4	162.7	172.4
1857	176.2	175.3	177.2	175.0	177.4
1858	202.5	194.8	210.2	192.6	212.3
1859	171.6	169.2	174.1	168.5	174.8
1860	172.5	170.3	174.7	169.7	175.3
1861	201.6	194.2	209.0	192.1	211.0
1862	150.6	141.2	160.0	138.6	162.6
1863	154.5	146.5	162.7	144.3	164.9
1864	166.9	162.9	170.9	161.8	172.1
1865	176.7	175.9	177.5	175.7	177.7
1866	166.6	162.6	170.8	161.4	171.9
1867	184.5	182.7	186.3	182.2	186.8
1868	202.7	194.9	210.4	192.7	212.6
1869	186.3	183.9	188.7	183.3	189.3
1870	192.6	188.1	197.0	186.9	198.2
1871	177.0	176.3	177.7	176.1	177.9
1872	184.4	182.6	186.1	182.2	186.6
1873	200.7	193.5	207.8	191.6	209.7
1874	173.3	171.4	175.2	170.9	175.8
1875	185.1	183.1	187.1	182.6	187.6
1876	190.1	186.4	193.7	185.4	194.7
1877	183.5	182.0	184.9	181.6	185.3

1878	173.8	172.0	175.5	171.6	176.0
1879	205.5	196.8	214.2	194.4	216.6
1880	186.2	183.8	188.5	183.2	189.1
1881	194.1	189.2	199.1	187.8	200.4
1882	209.9	199.7	220.0	196.9	222.7
1883	205.3	196.6	213.8	194.2	216.2
1884	199.4	192.7	206.1	190.8	207.9
1885	184.3	182.6	186.0	182.1	186.5
1886	200.4	193.3	207.3	191.4	209.2
1887	184.7	182.9	186.6	182.4	187.1
1888	196.7	190.9	202.5	189.3	204.1
1889	197.4	191.4	203.4	189.7	205.1
1890	189.3	185.9	192.6	185.0	193.6
1891	215.5	203.5	227.5	200.1	230.7
1892	173.7	171.9	175.5	171.4	176.0
1893	197.0	191.1	202.9	189.4	204.5
1894	246.5	224.2	268.6	218.0	274.6
1895	227.7	211.6	243.6	207.2	248.0
1896	209.9	199.7	220.0	196.9	222.8
1897	183.5	182.1	185.0	181.7	185.4
1898	181.2	180.5	181.9	180.3	182.1
1899	174.2	172.6	175.8	172.1	176.3
1900	151.4	142.3	160.6	139.8	163.1
1901	211.0	200.4	221.4	197.5	224.3
1902	171.8	169.4	174.2	168.7	174.9
1903	201.3	194.0	208.6	191.9	210.6
1904	204.8	196.3	213.2	193.9	215.5
1905	227.9	211.7	243.9	207.3	248.3
1906	211.2	200.6	221.7	197.6	224.6
1907	168.2	164.7	171.8	163.7	172.8
1908	185.2	183.2	187.2	182.6	187.7
1909	169.5	166.3	172.6	165.4	173.5
1910	156.3	148.8	163.8	146.7	165.9
1911	175.5	174.3	176.7	174.0	177.0
1912	195.5	190.1	200.8	188.6	202.3
1913	193.0	188.4	197.5	187.1	198.8
1914	179.3	179.2	179.4	179.2	179.4
1915	161.6	155.9	167.4	154.3	169.0
1916	197.2	191.2	203.2	189.6	204.8
1917	180.7	180.2	181.2	180.0	181.4
1918	192.8	188.3	197.3	187.0	198.5
1919	183.1	181.8	184.4	181.4	184.7
1920	178.9	178.8	178.9	178.8	179.0
1921	172.4	170.2	174.6	169.6	175.2
1922	186.8	184.3	189.4	183.6	190.1
1923	167.1	163.2	171.1	162.1	172.2
1924	198.6	192.2	205.1	190.4	206.8
1925	196.4	190.7	202.1	189.1	203.6

1926	190.5	186.7	194.2	185.7	195.2
1927	212.1	201.2	222.9	198.2	225.9
1928	162.4	156.9	167.9	155.4	169.4
1929	169.5	166.4	172.7	165.5	173.6
1930	214.8	203.0	226.5	199.7	229.7
1931	185.4	183.3	187.5	182.8	188.1
1932	175.6	174.5	176.8	174.1	177.1
1933	194.6	189.5	199.7	188.1	201.1
1934	205.7	196.9	214.4	194.5	216.8
1935	179.9	179.6	180.2	179.6	180.2
1936	184.3	182.6	186.0	182.1	186.4
1937	207.8	198.3	217.2	195.7	219.8
1938	184.1	182.4	185.7	182.0	186.2
1939	177.3	176.7	177.9	176.5	178.1
1940	165.5	161.1	170.0	159.9	171.3
1941	201.7	194.2	209.1	192.1	211.1
1942	175.5	174.3	176.7	174.0	177.0
1943	171.5	169.0	174.0	168.3	174.7
1944	183.4	182.0	184.8	181.6	185.2
1945	207.3	197.9	216.5	195.4	219.0
1946	186.3	183.9	188.7	183.3	189.3
1947	190.9	187.0	194.7	185.9	195.8
1948	190.2	186.5	193.8	185.5	194.8
1949	166.7	162.6	170.8	161.5	171.9
1950	182.0	181.1	183.0	180.8	183.3
1951	168.1	164.5	171.8	163.5	172.8
1952	169.6	166.5	172.8	165.7	173.6
1953	199.9	193.0	206.7	191.1	208.6
1954	177.6	177.0	178.1	176.9	178.2
1955	183.5	182.1	185.0	181.7	185.4
1956	170.3	167.4	173.2	166.6	174.0
1957	173.4	171.5	175.3	171.0	175.8
1958	159.8	153.4	166.2	151.7	167.9
1959	192.0	187.7	196.2	186.5	197.4
1960	192.2	187.8	196.5	186.7	197.6
1961	175.6	174.4	176.8	174.1	177.1
1962	158.3	151.5	165.2	149.7	167.1
1963	177.2	176.6	177.8	176.4	178.0
1964	188.1	185.1	191.0	184.3	191.8
1965	172.5	170.4	174.7	169.8	175.3
1966	186.3	183.9	188.7	183.3	189.3
1967	182.6	181.4	183.7	181.1	184.0
1968	164.1	159.2	169.1	157.8	170.4
1969	192.6	188.2	197.1	186.9	198.3
1970	197.2	191.2	203.1	189.6	204.8
1971	181.9	180.9	182.8	180.7	183.0
1972	201.6	194.1	209.0	192.1	211.0
1973	192.8	188.3	197.3	187.0	198.6

1974	170.4	167.6	173.3	166.8	174.1
1975	164.8	160.1	169.5	158.8	170.8
1976	192.2	187.9	196.5	186.7	197.7
1977	182.4	181.3	183.5	181.0	183.8
1978	186.2	183.8	188.5	183.2	189.2
1979	183.7	182.2	185.2	181.8	185.6
1980	198.4	192.0	204.7	190.3	206.5
1981	156.0	148.4	163.6	146.3	165.8
1982	161.9	156.2	167.6	154.6	169.1
1983	173.5	171.7	175.3	171.2	175.9
1984	160.3	154.1	166.5	152.4	168.2
1985	190.5	186.7	194.3	185.7	195.3
1986	182.4	181.3	183.6	181.0	183.9
1987	181.9	180.9	182.8	180.7	183.0
1988	177.6	177.1	178.1	176.9	178.2
1989	189.8	186.2	193.3	185.3	194.2
1990	173.3	171.4	175.2	170.9	175.8
1991	173.9	172.2	175.6	171.8	176.1
1992	181.4	180.6	182.1	180.4	182.3
1993	183.4	181.9	184.8	181.6	185.1
1994	187.5	184.7	190.3	184.0	191.1
1995	177.9	177.6	178.3	177.4	178.4
1996	171.6	169.1	174.1	168.4	174.7
1997	200.9	193.7	208.1	191.7	210.1
1998	175.1	173.8	176.4	173.4	176.8
1999	166.8	162.7	170.8	161.6	172.0
2000	164.6	159.8	169.4	158.5	170.7
2001	157.6	150.5	164.7	148.6	166.7
2002	178.2	178.0	178.5	177.9	178.6
2003	197.9	191.7	204.1	190.0	205.8
2004	189.4	186.0	192.8	185.0	193.7
2005	184.5	182.7	186.2	182.2	186.7
2006	182.5	181.4	183.7	181.1	184.0
2007	166.8	162.8	170.9	161.7	172.0
2008	155.4	147.6	163.2	145.5	165.4
2009	177.3	176.7	177.9	176.6	178.1
2010	182.2	181.2	183.2	180.9	183.5
2011	186.5	184.0	188.9	183.4	189.6
